# Hemoglobin–Albumin Cluster Incorporating a Pt Nanoparticle: Artificial O_2_ Carrier with Antioxidant Activities

**DOI:** 10.1371/journal.pone.0110541

**Published:** 2014-10-13

**Authors:** Hitomi Hosaka, Risa Haruki, Kana Yamada, Christoph Böttcher, Teruyuki Komatsu

**Affiliations:** 1 Department of Applied Chemistry, Faculty of Science and Engineering, Chuo University Tokyo, Japan; 2 Research Center of Electron Microscopy, Institute of Chemistry and Biochemistry Freie Universität Berlin, Berlin, Germany; Russian Academy of Sciences, Institute for Biological Instrumentation, Russian Federation

## Abstract

A covalent core–shell structured protein cluster composed of hemoglobin (Hb) at the center and human serum albumins (HSA) at the periphery, Hb-HSA_m_, is an artificial O_2_ carrier that can function as a red blood cell substitute. Here we described the preparation of a novel Hb-HSA*_3_* cluster with antioxidant activities and its O_2_ complex stable in aqueous H_2_O_2_ solution. We used an approach of incorporating a Pt nanoparticle (PtNP) into the exterior HSA unit of the cluster. A citrate reduced PtNP (1.8 nm diameter) was bound tightly within the cleft of free HSA with a binding constant (*K*) of 1.1×10^7^ M^−1^, generating a stable HSA-PtNP complex. This platinated protein showed high catalytic activities for dismutations of superoxide radical anions (O_2_^•–^) and hydrogen peroxide (H_2_O_2_), i.e., superoxide dismutase and catalase activities. Also, Hb-HSA*_3_* captured PtNP into the external albumin unit (*K* = 1.1×10^7^ M^−1^), yielding an Hb-HSA*_3_*(PtNP) cluster. The association of PtNP caused no alteration of the protein surface net charge and O_2_ binding affinity. The peripheral HSA-PtNP shell prevents oxidation of the core Hb, which enables the formation of an extremely stable O_2_ complex, even in H_2_O_2_ solution.

## Introduction

Hemoglobin (Hb)-based O_2_ carriers (HBOCs) have been studied extensively as a substitute for red blood cells (RBCs) in transfusion medicine and as O_2_ therapeutic reagents [1]–[5]. Nevertheless, none satisfies all requirements for use in clinical situations [6], [7]. A common side-effect is mild hypertension resulting from nitric oxide (NO) depletion by Hb diffused into the extravascular space [8], [9]. Actually, NO is an endothelial-derived relaxing factor. Moreover, HBOCs show faster autoxidation of Hb to the ferric heme form (metHb) than the native Hb shows [10]–[12]. Autoxidation of Hb produces a superoxide radical anion (O_2_^•–^), which is disproportionated to hydrogen peroxide (H_2_O_2_) [13]. These reactive oxygen species (ROS) promote the oxidation of Hb. In RBC, antioxidant systems include superoxide dismutase (SOD) and catalase, which catalytically scavenge O_2_^•–^ and H_2_O_2_, and thereby protect the Hb function. In ischemia-reperfusion when the ischemic tissue is reperfused with O_2_, xanthine oxidase converts xanthine and hypoxanthine into O_2_^•–^ [14]–[16]. Overproduction of O_2_^•–^ and subsequently H_2_O_2_ causes not only tissue injury, but also further oxidation of Hb. Consequently, in clinical situations involving ischemia-reperfusion, HBOC with antioxidant activity is expected to be tremendously useful. Chang et al. first synthesized polyHb-SOD-catalase conjugate and demonstrated the reduction of the autoxidation rate of Hb [17]. Kluger et al. reported that the metHb formation was inhibited in structurally defined Hb-SOD dimer [18]. Silaghi-Dumitrescu et al. prepared Hb copolymer with rubrerythrin, non heme iron enzyme [19]. These Hb-(antioxidant enzyme) conjugates displayed both O_2_ carrying and antioxidant properties. However, a specific enzyme is necessary to scavenge the individual ROS, and it denatures gradually.

**Figure 1:**
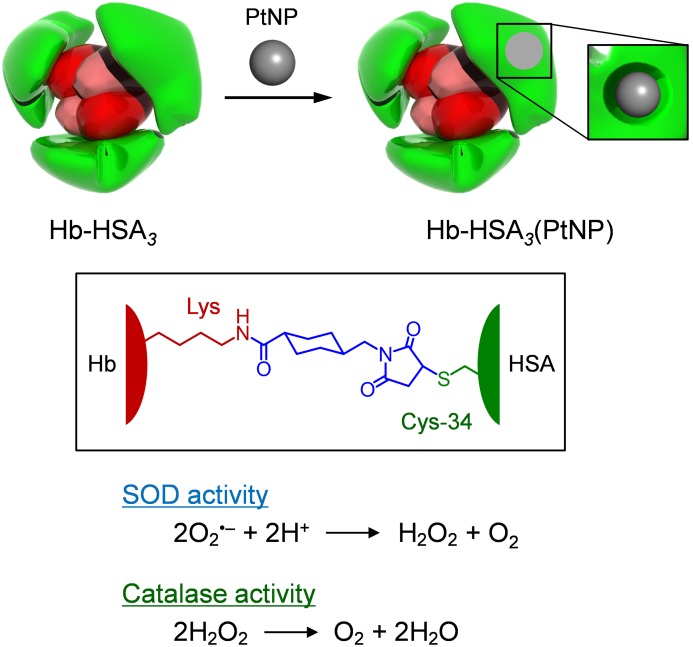
Schematic illustrations of Hb-HSA*_3_*(PtNP) cluster. The Cys-34 of HSA and the surface Lys group of Hb were connected covalently with a crosslinking agent (SMCC). A PtNP was bound within the cleft of the exterior HSA unit and performed SOD and catalase activities.

More recently, we synthesized a covalent core–shell structured protein cluster comprising Hb at the center and human serum albumins (HSA) at the periphery, Hb-HSA_m_ (m = 2, 3, 4), which acts as a unique HBOC (Figure 1) [20]. Since HSA contains only one cysteinyl thiol at position 34, we exploited a heterobifunctional crosslinker, *N*-succinimidyl 4-(*N*-maleimidomethy) cyclohexane-1-carboxylate (SMCC), as a connector between the Cys-34 residue of HSA and the surface lysyl *ε*-amino groups of Hb. The major product is the Hb-HSA_3_ heterotetramer in triangular form with an HSA-binding number (m) of three. HSA, the most prominent plasma protein, demonstrates low permeability in the vasculature walls because of the electrostatic repulsion between the negatively charged albumin surface [isoelectric point (*p*I): 5.0] and glomerular basement membrane around the endothelial cells [21]. From this physiological perspective, the surface net charge of the Hb-HSA_m_ cluster is satisfactorily negative (*p*I: 5.1–5.2) [20]. Intravenous transfusion of the Hb-HSA_m_ cluster is expected to enable long-term circulation without extravasation. Moreover, it might not elicit an unfavorable increase in blood pressure.

If one were able to confer antioxidant properties to the external HSA unit of Hb-HSA_m_, then this construct would become a promising O_2_ carrier with high resistance towards oxidation reactions. In this context, we chose Pt nanoparticle (PtNP) as a potential candidate. PtNPs have been widely investigated for a variety of applications, such as fine chemical synthesis, fuel cell fabrications, and biomedical treatments. It was reported that PtNP is an extremely effective catalysis for both O_2_^•–^ and H_2_O_2_ dismutations (Figure 1) [22]–[24]. (i) The high ROS scavenging activities of PtNP depend on greater surface area per mass relative to large particle [22], [23]. (ii) Almost no cytotoxicity was observed even after adherent cells were exposed to PtNPs [23]. We have found that small PtNP (1.8 nm diameter) is incorporated into HSA, and the obtained HSA-PtNP complex showed SOD and catalase activities with high efficiency. The Hb-HSA*_3_* also possesses the capability of binding PtNP into the exterior HSA shell. The resultant Hb-HSA*_3_*(PtNP) cluster forms a very stable O_2_ complex, even in aqueous H_2_O_2_ solution (Figure 1). This artificial O_2_ carrier, having triple functionalities (O_2_ transport, O_2_^•–^ dismutation, H_2_O_2_ dismutation) might be useful in clinical conditions with ischemia-reperfusion. The Hb-HSA*_3_*(PtNP) cluster would deliver O_2_ to the ischemic tissue, and simultaneously protect Hb and tissues from damaging effects of reperfusion injury.

## Materials and Methods

### Materials and apparatus

Human serum albumin (HSA) was purchased from Japan Blood Products Organization. Pure bovine Hb was purified from bovine blood purchased from Tokyo Shibaura Zouki Co., Ltd. [20]. Hydrogen hexachloroplatinate(IV) hexahydrate (H_2_PtCl_2_•6H_2_O), xanthine, and catalase (from bovine liver) were purchased from Wako Pure Chemical Industries Ltd. Ferricytochrome *c* (Cyt. *c*, from bovine heart) was purchased from Sigma-Aldrich Co. Xanthine oxidase (XOD, from butter milk) was purchased from Oriental Yeast Co., Ltd. Mn(III)-terakis(*N*-methylpyridinium) porphyrin (Mn-TMPyP) was purchased from Frontier Scientific Corp. Other chemicals of special grades were used without further purification. The water was deionized (18.2 MΩcm) using water purification systems (Elix UV and Milli Q Reference; Millipore Corp.). Isoelectric focusing (IEF) was performed using an electrophoresis power supply (EPS 601; GE Healthcare UK Ltd.) with an IEF gel (Novex pH 3–10; Invitrogen Corp.). The protein marker used was an IEF calibration kit Broad pI (pH 3–10; GE Healthcare UK Ltd.).

### Synthesis of PtNP

The citrate-reduced PtNP was prepared as described in a report of a study by Bond et al. [25]. To the refluxed aqueous H_2_PtCl_2_•6H_2_O solution (271 µM, 85.5 mL), 1 wt% trisodium citrate dihydrate in water (4.5 mL) was added and then refluxed continuously for 1 h with stirring. The solution changed to dark brown. After cooling slowly to 25°C, the obtained PtNP solution was washed with water using an ultrafilter (Q0100, 10 kDa MWCO; Advantec Toyo Kaisha Ltd.) in an UHP-76K ultraholder. Finally, the medium was concentrated up to 50 µM as PtNP using the UHP-76K ultraholder. The resultant PtNP colloid solution was stored in a refrigerator at 4°C.

### Preparation of Hb-HSA*_3_* cluster

The Hb-HSA*_3_* cluster was prepared according to our previously reported procedure with some modifications [20]. Typically, a DMSO solution of heterobifunctional crosslinker, *N*-succinimidyl 4-(*N*-maleimidomethy)cyclohexane-1-carboxylate (SMCC; Tokyo Chemical Industry Co., Ltd.) (20 mM, 4 mL) was added dropwise into phosphate buffered saline (PBS) solution (pH 7.4) of carbonyl Hb (0.1 mM, 40 mL), and the mixture was stirred for 3 h in the dark at 4°C. After removing unreacted crosslinker by gel filtration chromatography (GFC) with a Sephadex G25 (superfine) column, the obtained SMCC-bound Hb (maleimide activated Hb) was concentrated to 40 mL ([Hb] = 0.1 mM) using a centrifugal concentrator (Vivaspin 20 ultrafilter, 10 kDa MWCO; GE Healthcare UK Ltd.). Then this solution was added slowly into the PBS solution of HSA (1 mM, 40 mL) with subsequent stirring under dark conditions for 14 h at 4°C. A part of reaction mixture was applied to size-exclusion chromatography (SEC) on an HPLC system (LaChrom Elite; Hitachi High-Technologies Corp.) with a Shodex Protein KW-803 column (Showa Denko K.K.) using phosphate buffer (PB, pH 7.4, 50 mM) as the mobile phase. The elution curve exhibited new multiple peaks at the high molecular weight region. The three major components were identified as Hb-HSA_4_ heteropentamer (minor), Hb-HSA_3_ heterotetramer, and Hb-HSA_2_ heterotrimer [20]. Then the resultant solution was subjected to GFC with a Superdex 200 pg in XK50/60 column (GE Healthcare UK Ltd.) using PBS (pH 7.4) as the running buffer. We collected all major fractions before the HSA peak. The unreacted free HSA was excluded completely. By Hb and total protein assays [20], the average HSA/Hb ratio of the harvested Hb-HSA*_m_* cluster was found to be 2.8–3.2, which is indicated as Hb-HSA*_3_*. Finally, the obtained Hb-HSA*_3_* solution was condensed ([Hb] = 5 g/dL) using a Vivaspin 20 ultrafilter (30 kDa MWCO) and stored in a refrigerator at 4°C.

### CD measurements

Circular dichroism (CD) spectra were obtained using a spectropolarimeter (J-820; Jasco Corp.). The sample concentration was 0.2 µM in PBS. Quartz cuvettes with 10-mm thickness were used for measurements of 200−250 nm.

### Preparation of HSA-PtNP complex and Hb-HSA*_3_*(PtNP) cluster

The medium of PtNP solution was exchanged to PBS (pH 7.4) using a Vivaspin 20 ultrafilter (10 kDa MWCO). A PBS solution of HSA (0.51 mM, 0.1 mL) was added slowly to the PtNP solution (10.2 µM, 5 mL, PBS), and the mixture was incubated for 1 h with gentle stirring in the dark at 25°C, yielding HSA-PtNP complex (PtNP/HSA = 1/1). Similarly, the Hb-HSA*_3_* solution (0.51 mM, 0.2 mL, PBS) was added to the PtNP solution (10.2 µM, 10 mL, PBS). Then the mixture was incubated for 1 h with gentle stirring in the dark at 25°C, affording Hb-HSA*_3_*(PtNP) cluster (PtNP/Hb-HSA*_3_* = 1/1).

### Determination of binding constants of PtNP for HSA and cluster

Binding constants (*K*) of PtNP for HSA and Hb-HSA*_3_* cluster were determined using fluorescence quenching measurements of albumin by PtNP titration according to the literature [26]. Fluorescence of the HSA or Hb-HSA*_3_* ([HSA unit] = 10 µM) (*E*_m_: 340 nm) solution (PBS, pH 7.4) was quenched upon binding of PtNP (0–0.3 µM). The plots of *log*(*F*_o_–*F*)/*F* vs. *log*[PtNP] were produced from the data to obtain the *K* values and binding number.

### TEM measurement

Droplets of HSA-PtNP ([protein] = 0.35 mg/mL) were applied to amorphous carbon film covered 200-mesh grids (Quantifoil R1/4 with a hole diameter of approximately 1 µm; Quantifoil Micro Tools GmbH, Jena, Germany), which had been hydrophilized before use by plasma treatment (8 W, 60 s) in a Baltec Med 020 device (Leica Microsystems). After the supernatant fluid was blotted with a filter paper, an aqueous uranyl acetate (1 w/v %) was applied for another 45 s and the grids were eventually left to air-dry after blotting. Then the grids were transferred into a transmission electron microscope (Tecnai F20 microscope equipped with field emission gun operated at a 160 kV accelerating voltage; FEI Co.). Images were recorded using a CCD camera (Eagle 4k-CCD device; FEI Co.) operated at a binning factor of 2 (2,048×2,048 pixel).

### O_2_^•–^ scavenging activity (xanthine–XOD–Cyt. *c* assay)

O_2_^•–^ scavenging activity (SOD activity) of the HSA-PtNP complex was determined using the Cyt. *c* reduction technique, in which O_2_^•–^ was produced in situ by a xanthine–XOD reaction [27], [28]. The experiments were performed according to our previously reported procedure [29]. To the PB solution (pH 7.8, 50 mM, 3.0 mL) containing Cyt. *c* (10 µM), xanthine (50 µM), and catalase (500 U/mL) in a 10-mm path length optical quartz cuvette, an amount of XOD sufficient to give an initial rate of Δ*A*_550_ = 0.025 min^−1^ (without HSA-PtNP complex) (approximately 2.0 mU/mL) was injected at 25°C. After the addition of XOD, increases in the absorption at 550 nm based on the reduced-form Cyt. *c* was monitored at 25°C. From the absorbance increase, the initial rate constant (*v*_i_) was determined at various concentration of HSA-PtNP complex. The IC_50_ value is defined as the 50% inhibition concentration of Cyt. *c* reduction. The same experiments were also conducted for PtNP and HSA.

### H_2_O_2_ scavenging activity (quantitative peroxide assay)

H_2_O_2_ scavenging activity (catalase activity) of the HSA-PtNP complex was evaluated by measuring the concentration of residual H_2_O_2_ using the Pierce Quantitative Peroxide Assay Kits (Thermo Fisher Scientific Inc.). The HSA-PtNP solution (50 µM, 41 µL) was added to the aqueous solution of H_2_O_2_ (102 µM, 2.0 mL) in a vial bottle. Then the mixture was incubated with gentle stirring at 25°C. The 50 µL sample was pipetted out regularly from the reaction mixture and HSA-PtNP was removed using a centrifugal filter device (Microcon Ultracel YM-30; Millipore Corp.). Then 20 µL of the filtrate was mixed with the working reagent (200 µL) in a hole of a 96-well cell culture plate. The absorbance at 555 nm based on the (xylenol orange)-Fe(III) complex was measured using a Microplate Reader (iMark; Bio-Rad Laboratories, Inc.). From absorption at 550 nm, the concentration of residual H_2_O_2_ in the sample was determined using the calibration line ([H_2_O_2_] = 0–100 µM) prepared in advance. The *T*_50_ value is defined as time required for quenching half of H_2_O_2_. The same experiments were also conducted for PtNP, HSA, catalase, and Mn-TMPyP.

### O_2_ binding property

The visible absorption spectra of deoxy (under N_2_), oxy (under O_2_), and carbonyl (under CO) forms of the Hb-HSA*_3_* and Hb-HSA*_3_*(PtNP) clusters ([Hb]: 10 µM, PBS, pH 7.4) were obtained in accordance with our previously reported procedures using a UV–Visible spectrophotometer (8543; Agilent Technologies Inc.) equipped with a temperature control unit (89090A; Agilent Technologies Inc.) [20]. The O_2_ affinity (*P*_50_: O_2_-partial pressure where Hb is half-saturated with O_2_) and Hill coefficient (*n*) were determined using an automatic recording system for O_2_-equilibrium curve (Hemox Analyzer; TCS Scientific Corp.) using PBS (pH 7.4) at 37°C. The sample was oxygenated by an increasing O_2_-partial pressure and deoxygenated by flushing with N_2_.

### O_2_ complex stability

The O_2_ complex stability of the Hb-HSA*_3_* cluster was evaluated using the first-order autoxidation rate constant (*k*_ox_) of the central Hb. The PBS solution (pH 7.4) of oxyHb-HSA*_3_* cluster ([Hb] = 10 µM, 2 mL) was put into a 10-mm-path length optical quartz cuvette. The top of the cuvette was sealed with a gas permeation film (AeraSeal Film MAF710; Gel Co.), which allows air exchange and which prevents water evaporation. The absorption intensity at 630 nm (*A*_t_) based on metHb formation was monitored under aerobic conditions at 37°C. After the measurement, the entirely oxidized metHb-HSA*_3_* cluster was prepared by addition of slightly excess K_3_[Fe(CN)_3_], and its absorption intensity (*A*_100_) was observed. From the absorbance increase, the *k*_ox_ value was ascertained using nonlinear least-squares curve fitting techniques. The same experiments were conducted for native Hb and Hb-HSA*_3_*(PtNP) cluster.

The O_2_ complex stability of the cluster in 20 µM H_2_O_2_ solution was evaluated by the time course of metHb formation level because the mechanism of the Hb oxidation was complicated. To the PBS solution (pH 7.4) of oxyHb-HSA*_3_* cluster ([Hb] = 10 µM, 2 mL) in a 10-mm-path length quartz cuvette, aqueous H_2_O_2_ (2 mM, 20 µL) was added, and the absorption intensity at 630 nm (*A*_t_) was measured under aerobic conditions with gentle stirring for 180 min at 25°C. The top of the cuvette was sealed with a gas permeation film. After the measurement, a slightly excess K_3_[Fe(CN)_3_] was added to determine the absorption intensity of the entirely oxidized metHb form (*A*_100_). From the absorbance increase, the metHb level [(*A*_t_–*A*_0_)/(*A*_100_–*A*_0_)×100 (%)] (*A*_0_: absorption intensity at 630 nm before H_2_O_2_ injection) was ascertained. The same experiments were carried out for native Hb, Hb-HSA*_3_*(PtNP) cluster, and simple mixture of Hb/HSA-PtNP/HSA (1/1/2, molar ratio).

## Results and Discussion

### Synthesis and structure of HSA–PtNP complex

Enzymatic activities of PtNP have attracted considerable attention because of their potential applications for medical use [22]–[24]. Shirahata et al. reported high O_2_^•–^ and H_2_O_2_ dismutation activities of PtNPs and the highest enzyme reactivity at a particle size of about 2.0 nm [23]. In the circulatory system, the small PtNP (ca. 2 nm diameter) might be captured by HSA. However, the enzymatic properties of such postulated HSA-PtNP complex have not been reported in the relevant literature. We have now prepared the HSA-PtNP complex and have examined its O_2_^•–^ and H_2_O_2_ dismutation activities.

**Figure 2:**
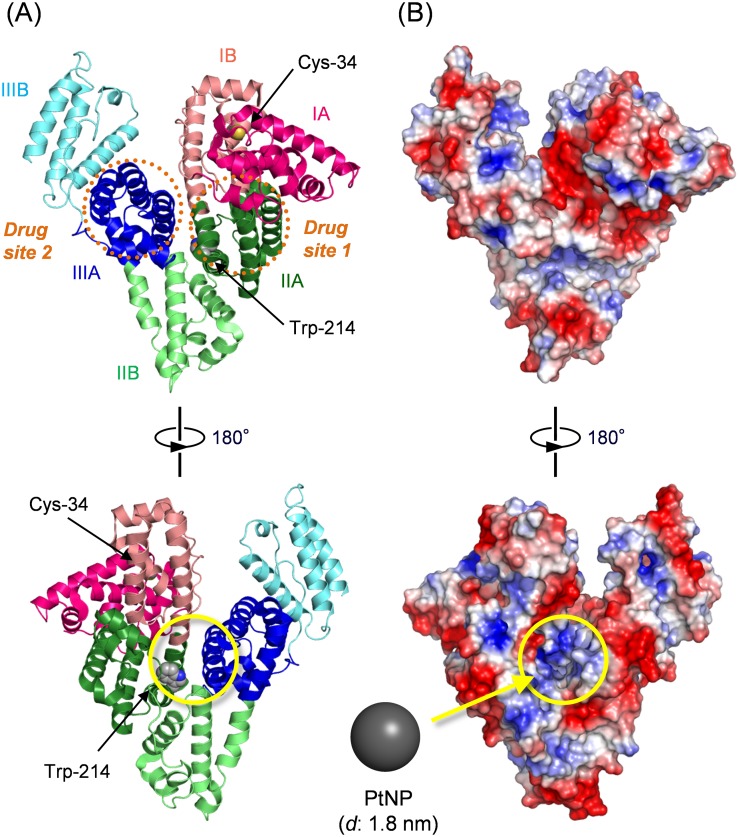
Crystal structure of HSA (PDB 1E78, ref. 31) and the PtNP binding site. (A) HSA structure involving the positions of drug site 1 (subdomain IIA, dark green), drug site 2 (subdomain IIIA, dark blue), Cys-34, and Trp-214. Cys-34 and Trp-214 are depicted in space-filling representation. The upper image and lower images respectively show the “front side” and “back side”. (B) Surface electrostatic potential representations of HSA in the same orientations illustrated in (A). Blue and red respectively represent positive charge and negative charge density. Possible binding site of PtNP in the positively charged cleft between subdomain IIA and IIIA is indicated by a yellow circle. These images were produced based on crystal structure coordinates using PyMOL (Schrödinger K. K., CA, USA).

HSA is a heart-shaped monomeric protein (66.5 kDa) consisting of three homologous domains (I–III), each of which contains two subdomains: A and B (Figure 2A) [30], [31]. Many water insoluble metabolites (fatty acids, bilirubin, thyroxin, etc.) and commonly used drugs (warfarin, diazepam, ibuprofen, etc.) bind to the principle ligand binding sites in subdomain IIA and IIIA of HSA: so-called drug sites 1 and 2 [32]. To embed a PtNP into this protein interior, we prepared citrate-reduced PtNP with a diameter of 1.5–2.0 nm [25]. TEM images clearly showed the formation of uniform PtNPs with diameter (*d*) of 1.8 nm. The PtNP concentration was calculated as 1.25 µM based on the Pt^2+^ concentration and particle size. The resultant aqueous PtNP solution was concentrated up to 50 µM using an ultrafiltration device. The medium was exchanged to phosphate buffered saline (PBS, pH 7.4). No precipitation was found for over one year at 4°C.

**Figure 3:**
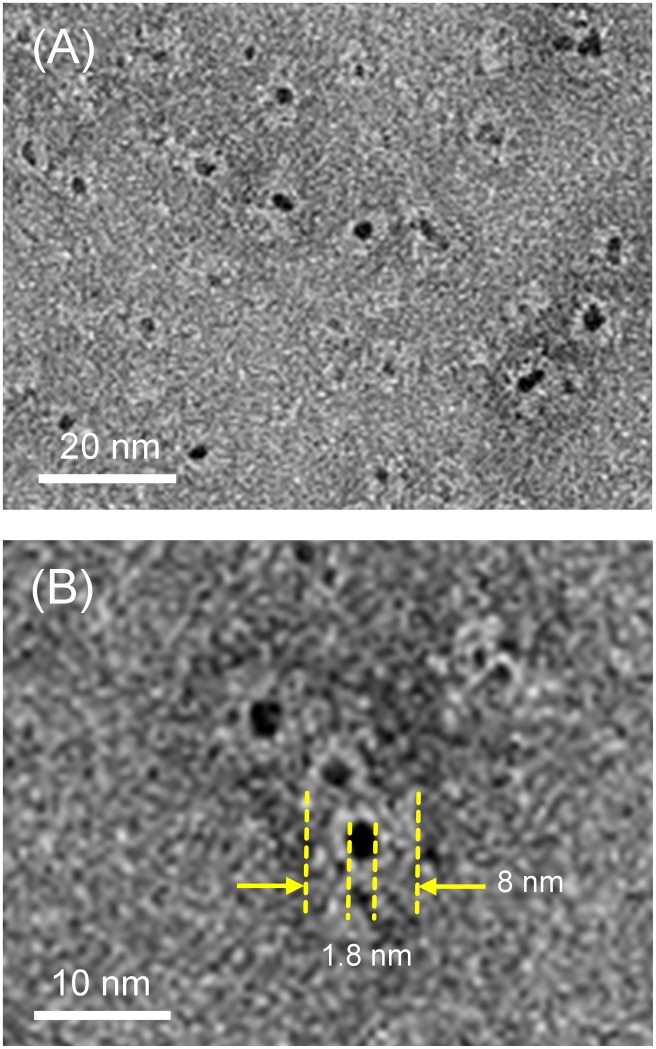
TEM images of HSA-PtNP complexes. The sample was negatively stained with 1% uranyl acetate.

The complexation of PtNP to HSA was conducted by adding HSA to the PtNP solution (PtNP/HSA = 1/1). Gel permeation chromatography (Sephadex G25) of the obtained protein displayed a single elution peak. Notably, TEM images demonstrated the formation of equivalent complex of HSA and PtNP (Figure 3A). Close inspections of TEM micrographs revealed that each PtNP is accommodated in the center of the protein (Figure 3B). One feasible binding mode is a covalent linkage between the thiol residue (Cys-34) of HSA and the PtNP surface. Nevertheless, nonmercapt HSA, in which Cys-34 is oxidized, also formed a similar HSA-PtNP complex, indicating that the covalent S-Pt bond is unlikely. Another possible binding force is electrostatic attraction between the negatively charged surface of PtNP and a positively charged region of the protein. Based on the electrostatic potential representation of HSA, we found a positively charged cleft between subdomain IIA and IIIA (Figure 2B). In fact, the fluorescence emission intensity of the HSA solution (*λ*_em_: 340 nm) was quenched by addition of PtNP. It is caused primarily by an energy transfer from the tryptophan (Trp)-214 residue in subdomain IIA (Figure 2A) to the bound PtNP. From titration measurements [26], the binding constant (*K*) and binding number of PtNP with HSA were calculated respectively as 1.1×10^7^ M^−1^ and 1.1. We reasoned that one PtNP binds to the positively charged cleft of HSA on the back side, yielding a 1∶1 HSA-PtNP complex. The obtained dark-brown protein solution was stable over one year at 4°C.

### Antioxidant activities of HSA–PtNP complex

The SOD activity of the HSA-PtNP complex was evaluated in phosphate buffered (PB) solution using the xanthine–(xanthine oxidase)–ferricytochrome *c* (Cyt. *c*) assay [27]–[29]. In the presence of the HSA-PtNP complex, the Cyt. *c* reduction by O_2_^•–^ was inhibited significantly. The IC_50_ value (the concentration of enzyme necessary to attain 50% inhibition of the Cyt. *c* reduction) of the HSA-PtNP complex was determined to be 0.16 µM (Table 1). Under our experimental conditions, the reduction of Cyt. *c* was not suppressed by HSA alone. For that reason, SOD activity of the albumin protein is excluded. The IC_50_ of HSA-PtNP complex is smaller than that of the best synthetic SOD model Mn(III)-tetrakis(*N*-methylpyridinium)porphyrin (Mn-TMPyP) [29] and resembled the value of native Cu,Zn-SOD [33]. We infer that the HSA-PtNP complex possesses a strong capability to catalyze the dismutation of O_2_^•–^.

**Table 1:** O_2_^•–^ scavenging activity (IC_50_) and H_2_O_2_ scavenging activity (*T*_50_) of HSA-PtNP complex at 25°C.

Enzyme mimics	IC_50_ (µM)^a^	*T*_50_ (min)^b^
HSA	N.D.	N.D.
PtNP	0.12	6
HSA-PtNP	0.16	19
Mn-TMPyP	0.8^c^	N.D.
Cu,Zn-SOD	0.03^d^	–
Catalase	–	≈0.1

a In PB solution (pH 7.8, 50 mM).

b In PBS solution (pH 7.4), [H_2_O_2_] = 0.1 mM.

c Ref. 29.

d Ref. 33. In PB solution (pH 7.8, 45 mM).

**Figure 4:**
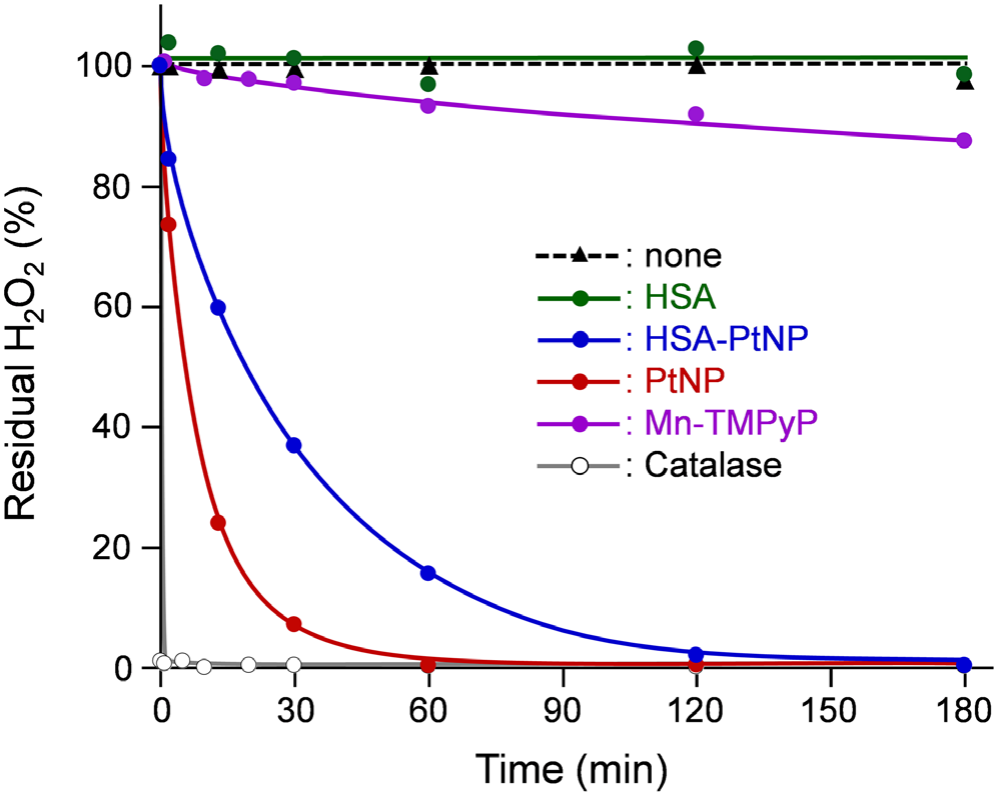
Time course of residual H_2_O_2_ percentage in 0.1 mM H_2_O_2_ solution with HSA-PtNP complex. [Sample] = 1 µM at 25°C.

Next, the catalase activity of the HSA-PtNP complex was examined by measuring the H_2_O_2_ decomposition. In the presence of HSA-PtNP, the H_2_O_2_ concentration declined considerably and reached zero after 180 min (Figure 4). The *T*_50_ value (time required for quenching half of H_2_O_2_) of HSA-PtNP was 19 min (Table 1). On the one hand, with the coexistence of HSA alone, the concentration of H_2_O_2_ was not changed. These results imply that the catalase activity of HSA-PtNP complex was based on the PtNP in the protein. While the *T*_50_ value is at least two order of magnitude larger than that of native catalase, this platinated protein showed much higher H_2_O_2_ dismutation activity than Mn-TMPyP [34]. Overall, we concluded that the HSA-PtNP complex shows strong abilities to catalyze the dismutation of both O_2_^•–^ and H_2_O_2_.

### Synthesis and structure of Hb-HSA*_3_*(PtNP) cluster

**Figure 5:**
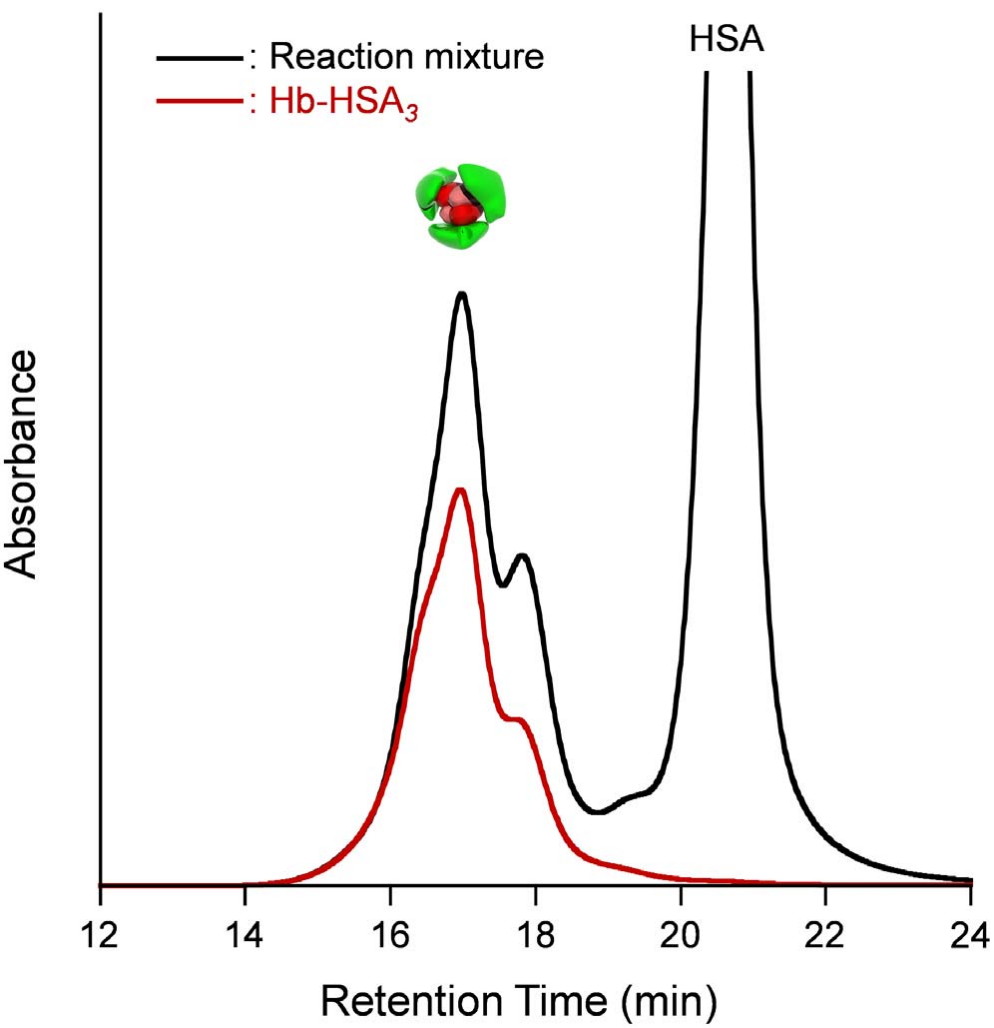
SEC profile of Hb-HSA*_3_* cluster. Black line: reaction mixture of SMCC-bound Hb and HSA, red line: separated Hb-HSA*_3_*.

**Figure 6:**
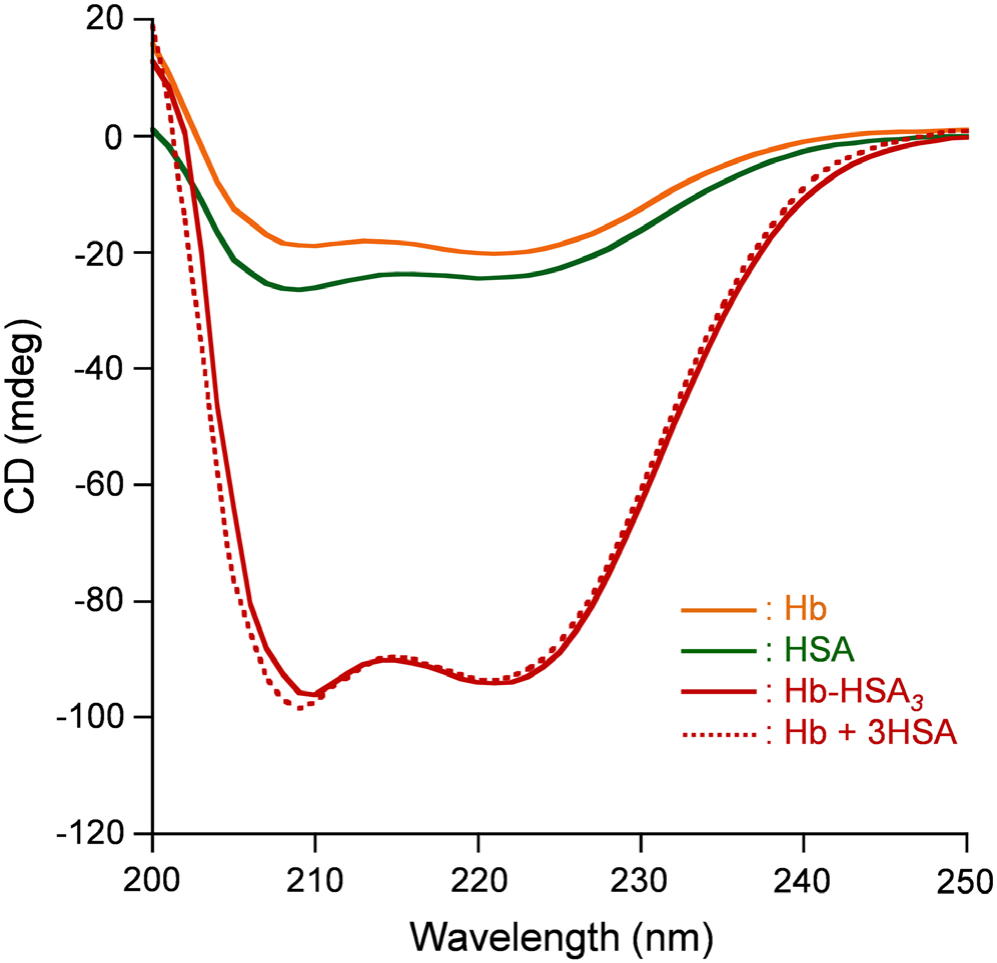
CD spectra of Hb, HSA, and Hb-HSA*_3_*. [Sample] = 0.2 µM in PBS solution (pH 7.4) at 25°C.

The Hb-HSA*_3_* cluster with the average HSA/Hb ratio of 3.0 was synthesized according to our previously reported procedure with some modifications (See Materials and Methods). Size exclusion chromatography (SEC) of the reaction mixture of SMCC-bound Hb and HSA exhibited new peaks of Hb-HSA_4_ heteropentamer (shoulder), Hb-HSA_3_ heterotetramer, and Hb-HSA_2_ heterotrimer (Figure 5); the major product was Hb-HSA_3_ (42%). By gel filtration chromatography (GFC), all the cluster fractions were harvested together (yield: 80% based on Hb). Unreacted free HSA was removed completely (Figure 5). The average HSA/Hb ratio was determined to be 2.8–3.2 using Hb and total protein assays. This protein cluster is shown as Hb-HSA*_3_*. The CD spectral pattern and intensity of the Hb-HSA*_3_* cluster agreed well with the sum of the Hb spectrum and a three-fold-enlarged HSA spectrum (Figure 6). This observation also supports the average HSA/Hb as 3 (mol/mol).

Then the Hb-HSA*_3_* solution was added slowly to the PBS solution of PtNP, yielding Hb-HSA*_3_*(PtNP) hybrid cluster (PtNP/Hb-HSA*_3_* = 1/1). From titration measurements [26], the *K* value and binding number of PtNP with the exterior HSA unit were ascertained as 1.1×10^7^ M^−1^ and 1.1, which are equal to the data observed for free HSA. The affinity of PtNP with HSA moiety of the cluster is satisfactorily high. Even though, PtNP may transfer to other plasma proteins after intravenous administration. To avoid such intermolecular exchanging reaction in vivo, covalent attaching of PtNP to the HSA unit would be beneficial. The isoelectric point (*p*I: 5.1) of Hb-HSA*_3_* was unaltered by PtNP incorporation. HSA has a high molecular surface net charge, thereby the *p*I value is known to be shifted slightly by ligand binding [35]. Thus, our result suggests that the PtNP is not adhered onto the HSA surface, but that it is embedded into the HSA shell.

### O_2_ affinity and O_2_ complex stability

**Figure 7:**
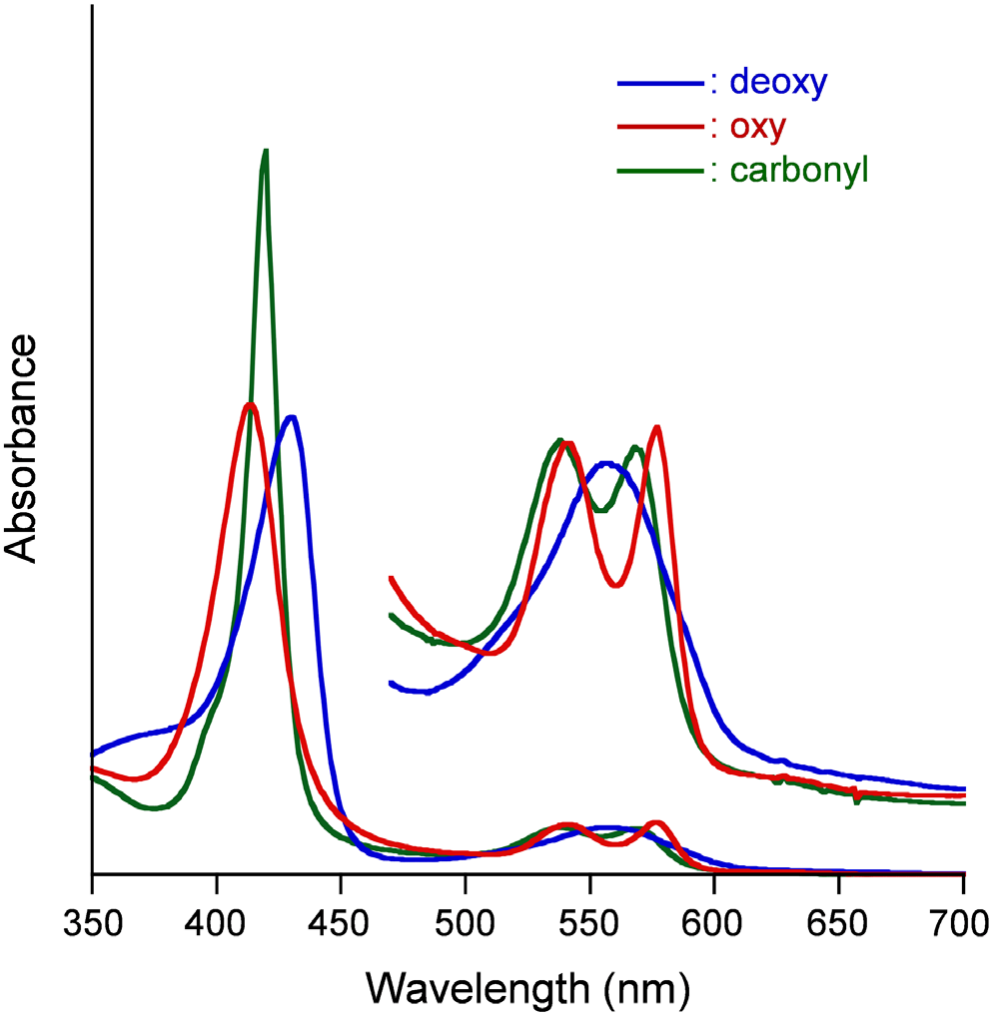
Visible absorption spectral changes of Hb-HSA*_3_* cluster. In PBS solution (pH 7.4) at 25°C.

The visible absorption spectral patterns of the Hb-HSA*_3_* cluster in PBS solution (pH 7.4) under N_2_, O_2_, and CO atmosphere (deoxy, oxy, and carbonyl forms) were fundamentally the same as those of Hb-HSA_3_ tetramer and native Hb (Figure 7, Table 2) [20], [36]. In contrast, the PBS solution of Hb-HSA*_3_*(PtNP) cluster exhibited strong absorbance over the entire visible range. It is ascribed to the superposing of the PtNP absorption onto the Hb-HSA*_3_* spectrum. Nevertheless, the absorption maxima of the Hb-HSA*_3_* and Hb-HSA*_3_*(PtNP) clusters showed good mutual agreement, indicating that PtNP caused no alternation of electronic states of the hemes in Hb (Table 2).

**Table 2:** Visible absorption spectral data of Hb-HSA*_3_* and Hb-HSA*_3_*(PtNP) clusters in PBS solution (pH 7.4) at 25°C.

	*λ*_max_ (nm)		
Hemoproteins	deoxy	oxy	carbonyl
Hb-HSA*_3_*	430, 556	413, 541, 577	420, 538, 569
Hb-HSA*_3_*(PtNP)	430, 554	413, 541, 576	419, 536, 568
Hb^a^	430, 555	414, 541, 577	420, 538, 569
HbA^b^	430, 555	415, 541, 577	419, 540, 569

a From ref. 20.

b HbA (human adult Hb), from ref. 36.

**Figure 8:**
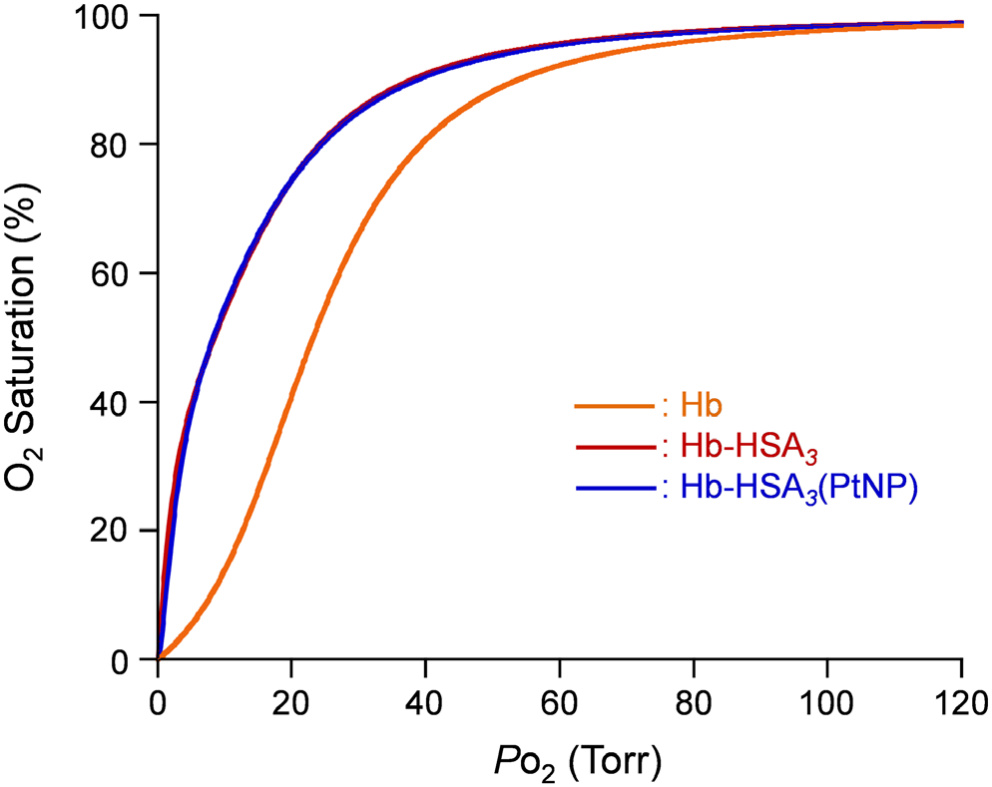
O_2_ equilibrium curves of Hb-HSA*_3_* and Hb-HSA*_3_*(PtNP) clusters. In PBS solution (pH 7.4) at 37°C.

The *P*_50_ (O_2_-partial pressure where Hb is half-saturated with O_2_) and cooperativity coefficient (Hill coefficient, *n*) of Hb-HSA*_3_* cluster (Figure 8, Table 3) were identical to the values of isolated Hb-HSA_3_ tetramer [20]. Moderate O_2_ affinity of Hb-HSA*_3_* cluster than native Hb might be attributable to the fact that the Cys-93(*β*) residue in Hb was blocked by the crosslinking agent SMCC and that Lys-82(*β*) was exploited as a binding partner of Cys-34 of HSA [20]. Nonetheless, the high O_2_ affinity might be favorable in application as a potential O_2_ carrier. Winslow et al. demonstrated that HBOC with a low O_2_ affinity engenders excessive O_2_ release in the arterioles and thereby invokes autoregulatory vasoconstriction [37], [38]. Intaglietta et al. reported that lower *P*_50_ (10 Torr) RBC provides improvement of microvascular function in comparison to the higher *P*_50_ (50 Torr) RBC in a hemorrhagic shocked hamster model [39]. In light of these investigations, the lower *P*_50_ might be effective to decrease arteriole O_2_ transport, potentially eliminating undesired cardiovascular side effects.

**Table 3:** O_2_ binding parameters of Hb-HSA*_3_* and Hb-HSA*_3_*(PtNP) clusters in PBS solution (pH 7.4) at 37°C.

Hemoproteins	*P*_50_ (Torr)	*n* (–)	*k*_ox_ (h^−1^)
Hb	23	2.6	0.037
Hb-HSA*_3_*	9	1.5	0.035
Hb-HSA*_3_*(PtNP)	9	1.5	0.039

Then the equilibrium between O_2_ and Hb-HSA*_3_*(PtNP) cluster was measured to investigate the effect of PtNP on the O_2_ affinity. The *P*_50_ and *n* values of the Hb-HSA*_3_*(PtNP) cluster were, respectively, 9 Torr and 1.5 (Figure 8, Table 3). The O_2_ binding parameters were unaffected by the PtNP association to the HSA shell. We inferred that the Hb-HSA*_3_*(PtNP) cluster retained two important benefits for RBC substitute: (i) negative surface net charge and (ii) high O_2_ affinity.

The O_2_ complex stability of the Hb-HSA*_3_*(PtNP) cluster in PBS (pH 7.4) was evaluated using the autoxidation rate constant (*k*_ox_) of the core Hb at 37°C. The *k*_ox_ value of native Hb was ascertained as 0.037 h^−1^; this result is well consistent with previously reported data [10], [40]. Remarkably, the Hb-HSA*_3_* cluster showed a similar *k*_ox_ (0.035 h^−1^) to that of native Hb. The oxyHb nuclei maintain high stability after conjugation with HSA. This fact contrasts with the fact that other HBOCs (PEGylated Hb, polymerized Hb, cross-linked Hb) show larger *k*_ox_ values relative to naked Hb [10]–[12]. A possible explanation of the stable O_2_ complex of our cluster is the enwrapping effect with HSA, which originally possesses a weak antioxidant property. As described earlier in this report, HSA itself showed no measurable SOD or catalase activities in our experimental conditions with a large excess amount of O_2_^–•^ and H_2_O_2_ (Table 1). Actually, HSA is known to be the predominant antioxidant in plasma (in vivo). Blache et al. estimated that 70% of the free-radical trapping activity of serum is attributed to HSA [41]. Otagiri et al. found that the antioxidant capabilities of HSA are attributable to the six methionine residues and Cys-34 [42]. Therefore, we inferred that covalent enwrapping with HSAs stabilizes the core Hb structure and affords a weak antioxidant effect to the hemes in Hb.

Unexpectedly, the *k*_ox_ value of Hb-HSA*_3_*(PtNP) cluster (0.039 h^−1^) was almost identical to those observed for Hb-HSA*_3_* and Hb. Kim et al. synthesized various protein-coated PtNPs and analyzed their ROS scavenging activities [24]. They demonstrated that O_2_^•–^ and H_2_O_2_ dismutation activities of the protein-coated PtNPs are greatly affected by the physicochemical properties and interior shape of the protein shells. In the Hb-HSA*_3_*(PtNP) cluster, the PtNP is bound to the cleft on the back side of HSA (Figure 2B), whereas the Cys-34 connection site to the Hb center is located on the front side of HSA. The accessibility of O_2_^•–^ and H_2_O_2_ from the Hb to PtNP in the HSA shell might be restricted because no accessible channel exists in the proteins.

**Figure 9:**
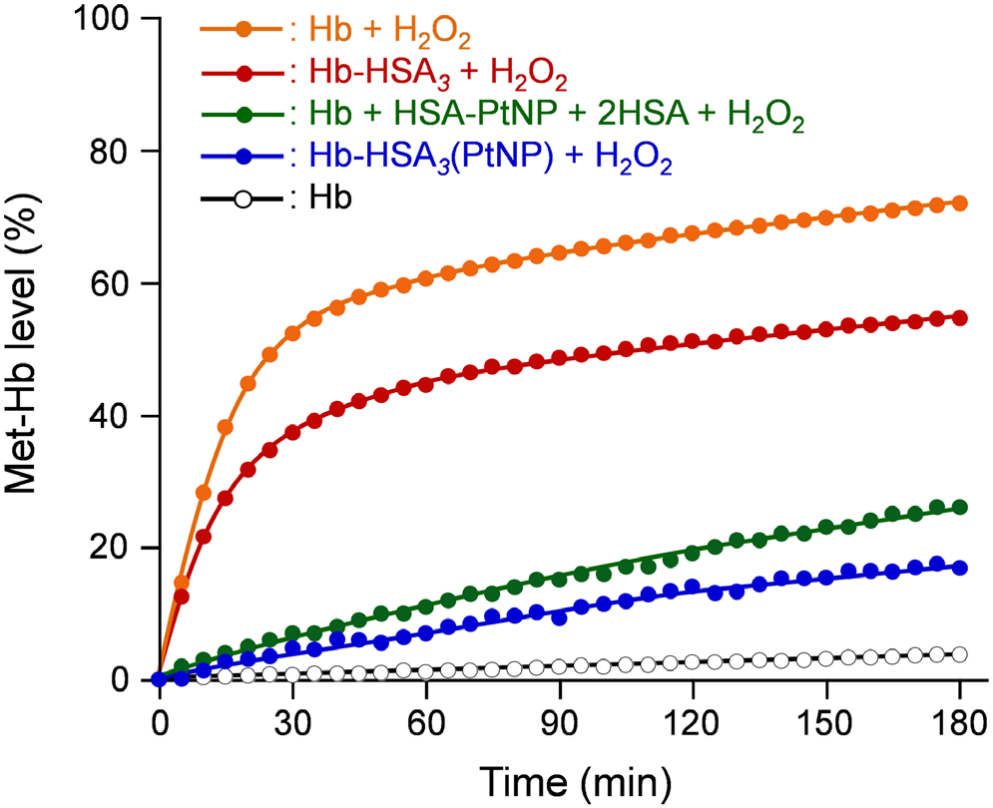
Time course of metHb level of Hb-HSA*_3_* and Hb-HSA*_3_*(PtNP) clusters. [Hb] = 10 µM in 20 µM H_2_O_2_ solution at 25°C.

Finally, we investigated the O_2_ complex stability of Hb-HSA*_3_*(PtNP) cluster in aqueous H_2_O_2_ solution. The H_2_O_2_ concentration in the human blood is assumed to be tens of micromolars (≤35 µM) [43]. Therefore, the oxidation rates of Hb-HSA*_3_*(PtNP), Hb-HSA*_3_*, and Hb in aqueous 20 µM H_2_O_2_ solution were examined. The time courses of the absorbance increase at 630 nm (which is due to metHb formation) were markedly different in these protein solutions (Figure 9). Native Hb showed a biphasic autoxidation curve. Approximately 50% Hb is oxidized rapidly in the initial phase within 30 min, followed by a second slow oxidation process. The metHb formation level reached 72% after 180 min. It is accepted that the *α* subunits in Hb are oxidized easily with respect to the *β* subunits [13]. Because the heme concentration was 40 µM ([Hb] = 10 µM), the *α* subunit oxidation occurred first, and subsequently the *β* subunits were oxidized.

The rate of metHb formation, however, was somewhat low in the Hb-HSA*_3_* cluster. In the initial phase, the metHb level increased to 37% within 30 min, followed by a slow oxidation reaction. This low rate appears to be attributable to a wrapping effect of HSA shell. As expected, the Hb-HSA*_3_*(PtNP) cluster was remarkably stable in H_2_O_2_ solution. We observed no initial oxidation process and only 17% metHb after 180 min, which is 24% of the value of native Hb. This result derives from the high antioxidant activity of the HSA-PtNP unit at the periphery. Actually the oxidation rate of Hb in the coexistence of HSA-PtNP and HSA (Hb/HSA-PtNP/HSA = 1/1/2), that are not covalently linked, was higher than that of the cluster. We can therefore conclude that the HSA-PtNP shell acts as an efficient scavenger for external H_2_O_2_ and achieves protection of the core Hb.

## Conclusion

A citrate-reduced PtNP (*d* = 1.8 nm) binds strongly within a cleft of HSA, generating a stable HSA-PtNP complex. This platinated protein showed high O_2_^–•^ and H_2_O_2_ dismutation activities. The Hb-HSA*_3_* cluster also captured PtNP into the external HSA unit. The obtained Hb-HSA*_3_*(PtNP) cluster formed an extremely stable O_2_ complex even in H_2_O_2_ solution. These results suggest that the Hb-HSA*_3_*(PtNP) cluster with (i) negative surface net charges, (ii) high O_2_ affinity, and (iii) antioxidant activities can be of tremendous medical importance as an alternative material to RBCs for transfusion in many clinical situations involving ischemia-reperfusion injury.
